# Antioxidant action of alpha lipoic acid on the testis and epididymis of diabetic rats: morphological, sperm and immunohistochemical evaluation

**DOI:** 10.1590/S1677-5538.IBJU.2018.0774

**Published:** 2019-09-02

**Authors:** Lanna Beatriz Neves Silva Corrêa, Carlos Alberto Soares da Costa, José Antônio Silva Ribas, Gilson Teles Boaventura, Mauricio Alves Chagas

**Affiliations:** 1 Departamento de Morfologia, Laboratório de Biomorfologia Celular e Extracelular, Universidade Federal Fluminense - UFF, Niterói, RJ, Brasil;; 2 Departamento de Nutrição e Dietética, Nutrição Experimental, Universidade Federal Fluminense - UFF, Niterói, RJ, Brasil;; 3 Departamento de Fisiologia e Farmacologia, Universidade Federal Fluminense - UFF, Niterói, RJ, Brasil

**Keywords:** Testis, Epididymis, Morphological and Microscopic Findings, Spermatozoa

## Abstract

**Introduction:**

Chronic hyperglycemia is caused by diabetes mellitus-committed genital morphophysiology, and oxidative stress is one of the main factors involved in this process. Alpha lipoic acid (ALA) can prevent metabolic and morphological changes in diabetic individuals.

**Objectives:**

In present study, we evaluated the effects of regular ALA consumption on the spermatogenesis and histoarchitecture in the male genital system of diabetic rats.

**Materials and Methods:**

Thirty-two Wistar rats were divided into groups: Control (CG); Diabetic Control (DCG), receiving commercial diet: ALA Group (ALAG) and Diabetic ALA Group (DALAG), fed diets with added ALA (300 mg/Kg bw). The diabetic groups received a single injection of streptozotocin (60 mg/kg). After sixty days of the diet, the animals were euthanized, and semen, testis and epididymis samples were collected. A histomorphometric analysis was performed to determine the epithelial height, tubular and luminal diameter, tubular and luminal area of seminiferous tubules and each epididymal region. Sertoli cells were evidenced using the antivimenti antibody and were quantified. The results were statistically analyzed by the ANOVA test.

**Results:**

At the end of the experiment, the DALAG glycemia was significantly lower than DCG. The histomorphometric parameters of the seminiferous and epididymal tubules did not show improvement in the DALAG. However, there was an improvement in the DALAG in terms of the concentration, motility and percentage of spermatic pathologies, as well as in the number of Sertoli cells (p<0.001).

**Conclusions:**

The results demonstrated that supplementation with the ALA antioxidant retards testicular lesions and preserve the process of spermatogenesis in diabetes.

## INTRODUCTION

Diabetes mellitus (DM) is a globally-known metabolic disorder characterized by chronic hyperglycemia and triggering systemic complications, among them cardiovascular diseases, nephropathies, and neuropathies (1, 2). The increased oxidative stress due to excess glucose in the body’s circulation results in a higher production of reactive oxidative species (ROS) or free radicals (3).

Testicular morphological changes as a consequence of the hyperglycemic effect of DM have been documented (4). The effect of DM on endocrine control of spermatogenesis is that it alters sexual gonads and their morphology (5). Decreased seminiferous and epididymal tubules, alterations in sperm parameters, decrease in Sertoli cell index, and damage in epithelial morphology with depletion and apoptosis of germ cells are associated with the resulting spermatogenesis impairment of oxidative stress (6, 7). The damage in the vimentin filaments of Sertoli cells causes dissociation of germ cells allowing apoptosis, which may result in sperm alterations in type I diabetes (8, 9).

Alpha lipoic acid (ALA) is known as a potent natural antioxidant with promising therapeutic applications, capable of restoring endogenous antioxidants (vitamins C and E), chelating free metals, and repairing oxidized proteins (10). It is used in the prevention of chronic conditions associated with oxidative stress, such as aging, cardiovascular diseases, as well as diabetes and its complications (11). ALA has been associated with improvement in the quality of life because it attenuates the symptoms of neuropathic diabetic patients who are given daily oral doses of the antioxidant (12).

Supplementation with antioxidants has been fundamental to prevent oxidative damage triggering reproductive dysfunction (13) and reverse testicular changes (14). The antioxidant effect of ALA on diabetes has been observed to be reflected in the decrease in glycemia levels (15). Mohasseb et al. (13) observed a reduction of oxidative damage in restoring the activity of the enzymes superoxide dismutase and glutathione peroxidase when ALA associated with vitamin C and vitamin E was administered, resulting in the improvement of testicular morphology.

Known for its potential in regenerating other antioxidants like vitamin C and vitamin E (16), ALA has been suggested as a therapeutic resource in dietary supplementation, whether associated with a normal diet or isolated oral supplement (17), reducing the oxidative damage of diabetes. The objective of this study was to evaluate the antioxidant effect of alpha lipoic acid in rats with STZ-induced diabetes on chronic hyperglycemia and its potential protective effect on spermatogenic dysfunction and male infertility (4, 13, 18).

## MATERIALS AND METHODS

### Experimental Protocol

The procedure with animals was approved by the Statistical Committee for the Use of Animals of the Federal Fluminense University (protocol CEUA-UFF number 799/16). All procedures followed the norms of the National Research Council (US) Institute for Laboratory Animal Research. Adult male rats of the *Rattus norvegicus albinus* variety, also known as Wistar, were kept in individual cages in the experimental animal room with relatively humid environments of 21–23ºC with 60% humidity, clear and dark cycle control, and water provided *ad libitum*.

### Animals

Thirty-two rats (12 weeks of age and weighing 250g) were randomly divided (n=8/group) into: Control Group (CG) and Diabetic Control Group (DCG) had a casein-based diet; ALA Group (ALAG) and Diabetic ALA Group (DALAG) were supplemented with ALA at a dose of 300 mg/kg body weight mixed with mash commercial feed.

### Streptozotocin treatment and induction of diabetes

Before receiving the experimental diets, 16 rats were separated and induced with diabetes with a single intraperitoneal injection of buffer (0.1 moL/L sodium citrate, pH 4.5) of streptozotocin (STZ, Sigma Aldrich S0130) at a dose of 60 mg/kg (19). Normal groups were given citrate buffers to be submitted to the same injection protocol. After 72 hours, diabetes was confirmed by the measurement of glycemic levels after a 5-hour fasting period, and animals that had plasma levels of 270 mg/dL or greater were considered in the experiment (13). The animals were divided to initiate treatment.

### Experimental Diet

The experimental diet was prepared in the Laboratory of Experimental Nutrition (LabNe) of UFF. The control groups consumed pelleted commercial feed (Nuvilab®, Nuvital, Paraná, Brazil) (Table-1). ALAG and DALAG consumed rations supplemented with 4-6g/kg ALA ration: purified R-isomer (Sigma Aldrich 62320). The ingredients were weighed and homogenized with boiling water in a Hobart® industrial mixer (São Paulo, SP, Brazil). The obtained mass was transformed into pellets and dried in a ventilated oven (Fabbe-Primar® nº 171, São Paulo, SP, Brazil) at 60ºC for 24 hours.

**Table 1:** Food composition of experimental groups.

Humidity (max)	125 g/kg
Crude protein (min)	220 g/kg
Ethereal extract (min)	40 g/kg
Mineral Matter (max)	90 g/kg
Gross Fiber (max)	70 g/kg
Calcium (min- max)	10-14 g/kg
Phosphorus (min)	8 g/kg
Alpha Lipoic acid*	4 – 6 g/kg
Vitamin A (min)	13.000 IU
Sodium (min)	2.7 g/kg
Iron (min)	0.05 g/kg
Manganese (min)	0.06 g/kg
Zinc (min)	0.06 g/kg
Copper (min)	0.01 g/kg
Iodine (min)	0.002 g/kg
Selenium (min)	5 x 10-5 g/kg
Cobalt (min)	0.0015 g/kg
Fluorine (max)	0.08 g/kg
Lysine (min)	12 g/kg
Methionine (min)	4 g/kg

Composition for each kg of food (minimum and / or maximum quantities). The groups consumed pelleted commercial feed (Nuvilab®, Nuvital, Paraná, Brazil) *Addition in Alpha Lipoic acid group and Diabetic Alpha Lipoic acid group.

### Glycemic Analysis

After confirmation of diabetes by STZ induction the weight and glycemia of all the animals was measured. Establishing the 5-hour fasting period, the blood was punctured through the caudal vein and analysed with the Accu-Check Perfoma (Glucometer®, Roche) glycometer (14) weekly until the end of the experiment.

### Sperm Parameters

After 60 days of consumption, the rats were euthanized with ketamine (75 mg/kg) along with xylazine (10 mg/kg). Immediately after the sacrifice, both testes and epididymis were collected. The tail of the right epididymis was sectioned and washed with 1mL of powdered milk thinner (20). Then a drop of this solution, placed on a heated blade at 35ºC, was covered with cover slip for evaluation of motility (percentage) and vigour (0-5). These parameters were observed through optical microscopy, following the protocol established by the Brazilian College of Animal Reproduction (21). Subsequently, the hyposmotic test was performed as proposed by Dell’Acqua et al. (22). Spermatozoa diluted in Kenney’s medium, (20) in a ratio of 1:100 in saline formalin, were taken to the Neubauer chamber to calculate the sperm concentration (million/mL). The spermatozoa were also evaluated for their morphology following the recommendation of Filler (23).

### Histological Processing

The left testicles and epididymis were separated. The testes were cleaved transversally and the epididymis longitudinally for visualization of the epididymal regions (6). The samples were fixed in Bouin’s solution and then stored in 10% buffered formalin. All material was included in the standard paraffin technique and 5 μm thick sections were subsequently stained with hematoxylin and eosin for histopathological examination and by immunohistochemistry for vimentin detection.

### Morphometric analysis of the seminiferous tubules and epididymal regions

Sections stained with hematoxylin and eosin, visualized on an optical microscope coupled to a digital camera, were obtained from analysis and quantification of the seminiferous and epididymal tubules. Fifty seminiferous tubules were captured after the selection of the most rounded tubules according to Leblond and Clermont’s criteria (24). From the epididymal tubules, 20 tubules from the head, body, tail were randomly captured. All images were scanned for total area (TA) and luminal area (LA) measurement, total (TD) and luminal diameter (LD), and epithelial height (EH), which were analysed using ImageJ® software (version 1.50g, National Institutes of Health, Bethesda, MD, USA).

### Histomorphometric Parameters

The tubule area was verified by delimiting the tubule in the basal membrane (TA) and bypassing the seminiferous tubule spermatids (LA). The TD and LD were measured using the average of two perpendicular lines. The EH was evaluated by the mean of four measurements in the epithelium (25).

### Immunohistochemical Processing

Immunolabeling for mapping Sertoli Cells with an anti-vimentin monoclonal antibody (mouse 1:100) (Clone v9, Dako) was associated with the EnVision FLEX development system using 4 μm thick histological slices on salinized slides. The cuts were submitted to the automated process of dewaxing, hydration, and antigenic recovery in a single step in the PTLink Dako PT100 equipment. Then, the cuts were circled by the DAKO S2002 hydrophobic pen to prevent the diluted antibody solution from flowing. Subsequently, the slides were dehydrated in alcohol with increasing concentrations and submitted to four xylol baths. The slides were assembled with ALLKIMIA Synthetic Canada Balm for further microscopic analysis.

### Evaluation of Sertoli Cells

The intermediate filaments of the Sertoli cells were labelled with vimentin and counted by the Cell Counter *plugin* of the Image J program. The mean number of cells counted per tubule of each experimental group was calculated according to the method proposed by Corrêa et al. (25).

### Statistical analysis

The results were statistically evaluated using the one-way ANOVA test associated with the Bonferroni multiple-comparison test in the GraphPad InStat® version 3.01 program. The graphs shown were performed on GraphPad Prism® version 5. The significance in all tests was set at the p<0.05 level.

## RESULTS

### Glycemia

To begin the experiment, the animals were induced with STZ and after 72 hours the increase in glycemia above 270 mg/dL determined the establishment of type 1 diabetes. At the end of 60 days, DCG presented significant elevation of hyperglycemia (P<0.001). Unlike DALAG, at week 8, hyperglycemia was not statistically significant compared to the beginning of the experiment. According to Table-2, the hyperglycemia presented at the 8th week by DCG was significantly higher than the glycemic increase presented by DALAG.

**Table 2:** – Glycemia, biometric of the testes and epididymis and histomorphometric analysis of the seminiferous tubules.

Glycemia (mg/dL)	CG	ALAG	DCG	DALAG	P value
After 72h	127.04±6.76	124.64±7.93	440.12±52.66 ^a, b^	429.66±62.68 ^a, b^	<0.0001
Last Day	118.33±9.07	138.33±17.78	576.50±30.13 ^a, b^	498.50±51.83 ^a, b, c^	<0.0001
**Parameters**					
Testicle weight (g)	1.77±0.11	1.81±0.15	1.62±0.41	1.39±0.34	0.0598
Epididymis weight (g)	1.03±0.08	1.31±0.33	0.57±0.33 ^a, b^	0.62±0.13 ^b^	<0.0001
Testicle + Epididymis Weight (g)	2.74±0.20	3.47±0.93^a^	2.19±0.54 ^a^	2.02±0.47 ^a^	0.0003
Epithelial Height (µm)	59.09±7.75	58.61± 3.61	23.44±2.41 ^a, b^	24.64±2.54 ^a, b^	0.0001
Total diameter (µm)	340.77±18.06	318.35±9.24	114.60± 27.15 ^a, b^	127.47± 26.22 ^a, b^	0.0886
Luminal diameter (µm)	208.33±38.84	205.33± 10.22	97.82± 8.58 ^a, b^	100.69± 2.16 ^a, b^	0.9054
Total area (µm^2^)	88.62x10^3^±59.11x10^2^	82.99x10^3^±53.06x10^2^	35.58x10^3^±28.11x10^2 a, b^	35.52x10^3^±19.74x10^2 a, b^	0.0001
Luminal area (µm^2^)	73.29 x10^3^±47.29x10^2^	69.37x10^3^±28.08x10^2^	30.25x10^3^±30.67x10^2 a, b^	31.82x10^3^±13.46x10^2 a, b^	0.0001

Data are presented as mean±s.d. Values obtained from ANOVA test. ^**a**^ = Statistically significant differences from GC; ^**b**^ = Statistically significant differences from ALAG; ^**c**^ = Statistically significant differences from DCG. **CG** = Control Group; ALAG - ALA Group; **DCG** = Diabetic Control Group; **DALAG** = Diabetic ALA Group.

### Testicular Histomorphometry

**Figure 1:**
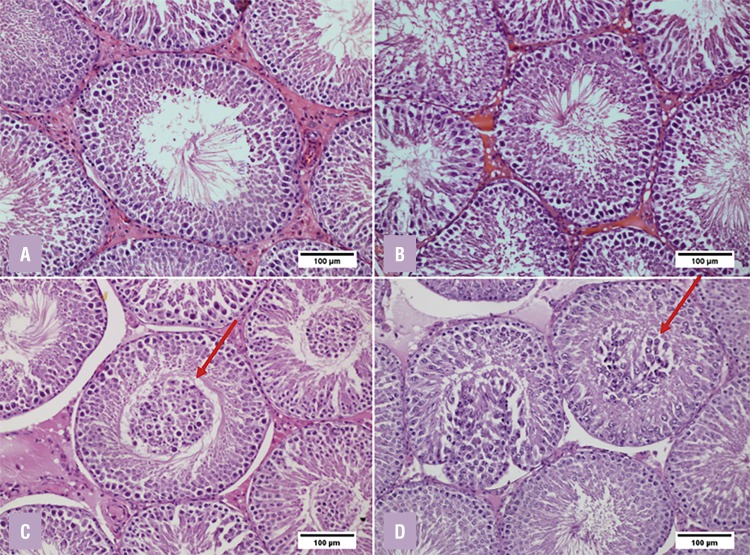
Photomicrographs comparing normal and diabetic groups. The arrows point to the epithelial flaking resulting in the agglomeration of the cells in the tubular lumen in the diabetic groups.

Observed in the STZ-induced groups, there was a marked decrease in tubular diameter, desquamation of germ cells, and agglomeration of cells in the tubular lumen (Figure-1). In the testicular histomorphometry of Table-2, the parameters showed tubular reduction of the diabetic groups compared to the control groups (P<0.001). The results showed that DALAG and DCG did not present significant differences between them.

**A** - Control Group; **B** - ALA Group; **C** - Diabetic Control Group; **D** - Diabetic ALA Group. (HE, 200x)

### Epididymal Histomorphometry

The groups induced by STZ presented a significant decrease compared to the control groups in the parameters of the total and luminal areas, including the total and luminal diameters (p<0.001) of all epididymal regions. There was no significant change in epididymal tubules between DALAG and DCG (Table-3).

**Table 3:** Histomorphometric analysis of epididymal tubules - head, body and tail.

Parameters	CG	ALAG	DCG	DALAG
**Head**				
TA (µm^2^)	32.77x10^3^±16.29x10^2^	29.83x10^3^±20.03x10^2^	25.97x10^3^±27.30x10^2 a, b^	26.26x10^3^±22.22x10^2 a^
LA (µm^2^)	16.86x10^3^±33.47x10^2^	15.16x10^3^±34.87x10^2^	10.22x10^3^±28.75x10^2 a, b^	11.96x10^3^±15.31x10^2 a^
TD (µm)	209.58±21.64	201.73±25.20	174.03±19.63^a, b^	180.17±8.94
LD (µm)	143.89±12.74	135.01±14.33	109.34±17.83^a, b^	120.78±9.53^a^
EH (µm)	32.15±5.76	30.91±6.75	30.49±2.90	28.34±2.35
**Corpus**				
TA (µm^2^)	99.98x10^3^±11.24x10^3^	95.53x10^3^±14.47x10^3^	74.95x10^3^±91.35x10^2 a, b^	70.36x10^3^±76.50x10^2 a, b^
LA (µm^2^)	76.91x10^3^±97.75x10^2^	74.59x10^3^±12.30x10^3^	53.42x10^3^±81.73x10^2 a, b^	49.81x10^3^±69.53x10^2 a, b^
TD (µm)	351.68±22.96	344.61±26.52	307.32±18.35 ^a, b^	294.63±17.61 ^a, b^
LD (µm)	307.77±20.80	300.78±25.11	255.37±19.24 ^a, b^	244.28±19.62 ^a, b^
EH (µm)	20.39±1.55	19.28±1.43	23.22±2.32 ^a,b^	22.90±1.49 ^b^
**Tail**				
TA (µm^2^)	23.09x10^4^±42.66X10^3^	28.53x10^4^±51.23x10^3^	13.88x10^4^±34.60x10^3 a, b^	97.25x10^3^±31.84x10^3 a, b^
LA (µm^2^)	20.34x10^4^±40.28x10^4^	24.87x10^4^±61.19x10^3^	10.73x10^4^±41.34x10^3 a, b^	73.13x10^3^±32.85x10^3 a, b^
TD (µm)	525.39±46.25	577.24±65.18	397.08±71.87 ^a, b^	339.85±60.18 ^a, b^
LD (µm)	493.44±46.42	543.16±69.27	354.89±76.48 ^a, b^	284.91±72.42 ^a, b^
EH (µm)	13.99±1.46	14.12±2.74	21.41±3.15 ^a, b^	25.95±3.42 ^a, b^

Data are presented as mean±s.d. Values obtained from ANOVA test. ^**a** =^ Statistically significant differences from GC; ^**b** =^ Statistically significant differences from ALAG; ^**c** =^ Statistically significant differences from DCG. **CG** = Control Group; **ALAG** = ALA Group; **DCG** = Diabetic Control Group; **DALAG** = Diabetic ALA Group; **TA** = Total area; **LA** = Luminal area; **TD** = Total diameter; **LD** = Luminal diameter; **EH** =- Epithelial height.

### Sperm Evaluation

**Figure 2:**
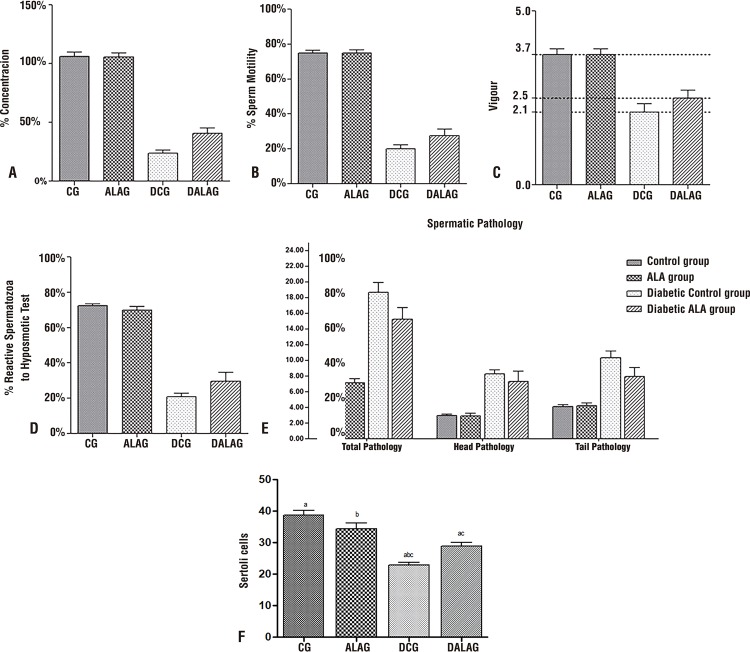
Sperm parameters.

According to Figure-2, the diabetic groups had lower means for concentration (Figure-2A), motility (Figure-2B), vigour (Figure-2C), and hyposmotic tests (Figure-2D). There was significant improvement in concentration and motility in the diabetic group supplemented with ALA as compared to the control diabetic group (p<0.0001). The sperm pathologies (total head and tail) were present in most diabetic groups. For this parameter, the group supplemented with ALA presented lower pathologies than DCG (p<0.0001).

Graph representing the averages of **A** - Concentration. **B** - Sperm Motility. **C** - Vigour. **D** - Hyposmotic Test and **E** - Spermatic Pathologies of the normal and diabetic groups after the supplementation of ALA. **F** - Count of Sertoli cells by seminiferous tubule after labeling with vimentin. DCG presented a reduced mean number of Sertoli cells compared to the other groups.

### Sertoli Cell Count

**Figure 3:**
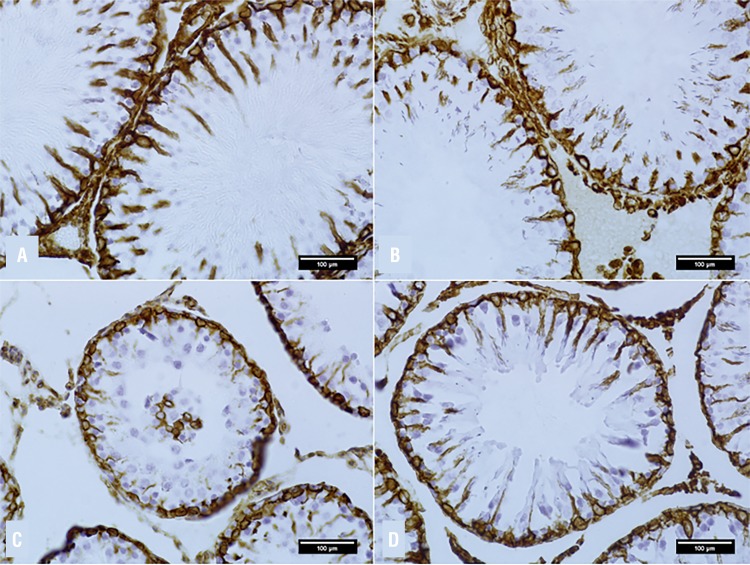
Photomicrographs of the Immunohistochemistry of Seminiferous Tubules marked by Vimentin allowing the cell count of Sertoli by tubule.

Sertoli cells were evidenced by the immunohistochemical labelling of vimentin (Figure-3). The diabetic groups had lower numbers of cells than CG and ALAG. In the cell count by tubules (Figure-2F), the number of cells in DALAG was significantly higher compared to DCG (p <0.001).

**A**- Control Group; **B**- ALA Group; **C**- Diabetic Group Control; **D**- Diabetic ALA Group. DAB chromogen. **Magnification A-B**: 400x; **C-D**: 200x.

## DISCUSSION

The induction of DM by streptozotocin was efficient in establishing pathological lesions affecting spermatogenesis. Chronic hyperglycemia is the main cause of increased production of ROS that favours tissue oxidative damage and may lead to sexual dysfunction (18). Antioxidants are known to reduce such tissue damage, especially in the diabetic setting (11-14).

Induction of diabetes by streptozotocin was confirmed in diabetic groups (>270mg/dL). Hyperglycemia was evaluated throughout the experiment and, at the end of 8 weeks, the supplemented rats presented an attenuation of the serum level compared to the first glycemic measurement. The diabetic group supplemented with ALA had lower final hyperglycemia than the control diabetic group. This data agrees with Mohasseb et al. (13) that showed that oral administration of ALA in combination with ascorbic acid and tocopherol promoted attenuation of glucose after 60 days of experiment. Alpha lipoic acid has the ability to restore other antioxidants, such as ascorbic acid and tocopherol to allow hypoglycemic action in diabetes (18).

Histopathological changes were observed and evidenced in testicular histomorphometry in diabetic groups after induction by streptozotocin caused by free radicals. Kaplanoglu et al. (19) submitted diabetic rats to the oral dose of vitamin E for 4 weeks and found that the testicular morphology altered by streptozotocin-induced diabetes did not improve and had satisfactory results with the association of green tea. Aguirre-Arias et al. (14), evaluating vitamin C alone, observed a reversal in the histological changes of the seminiferous tubules of diabetic rats supplemented for 63 days. Mohasseb et al. (13) evaluated glycemia reduction and protective effect on spermatogenic cells in diabetes-induced rats after oral administration of ALA associated with vitamins C and E. However, in our experiment, the alpha lipoic acid provided in the diet did not favour the improvement of testicular histomorphometric parameters.

Regarding epididymal regions, the histomorphometric analysis showed tubular decrease of all epididymal regions influenced by diabetes. Similar results were observed by Soudamani et al. (6), who reported changes in the different regions of the epididymis as a decrease in tubular and luminal diameter in diabetic rats. The oxidative stress of diabetes can inhibit steroidogenesis without modifying the gonadal histoarchitecture (26). The diet supplemented with ALA had no effect on epididymal morphology in the diabetic rats.

The damage of diabetes on sperm parameters is similar to previous studies (27). Alpha lipoic acid administered under conditions of oxidative stress improved the motility and concentration of the diabetic group, possibly through interaction with other antioxidants. Aguirre-Arias et al. (14) observed that the antioxidant ascorbic acid was able to reverse testicular damage, but the restoration of sperm motility or fertility was insufficient. ALA is capable of improving viability and sperm motility, minimizing DNA damage by its ability to penetrate the Krebs cycle, aiding in the production of ATP (28). Studies indicate that the use of ALA restoring spermatological parameters disturbed by the use of industrial substances improves sperm quality in cases of high testicular temperature and increased oxidative stress from varicocele (29, 30).

The expression of vimentin is found in Sertoli cells associated with membrane integrity. Xu et al. (8) observed a decrease of Sertoli cells in diabetics. This data was confirmed by our study. The group of diabetic rats supplemented with the ALA antioxidant presented higher numbers of cells, suggesting its effectiveness in protecting and delaying the apoptosis of Sertoli cells. It seems reasonable to suppose that this positive action on the Sertoli cells directly influences the parameters of the sperm evaluation of the diabetic group treated with ALA, which could explain the improvement observed in all parameters analysed.

## CONCLUSION

DM is a disease known worldwide for compromising reproductive physiology. Our results suggest that oral ALA supplementation attenuates the loss of Sertoli cells and improves the concentration and sperm motility affected by diabetes.
